# Bridging the gaps in inclusive palliative care: Reflections on a socioecological perspective for LGBTQIA + populations

**DOI:** 10.1017/S147895152510134X

**Published:** 2025-12-29

**Authors:** Andika Ari Saputra, Dita Kurnia Sari

**Affiliations:** 1 Department of Guidance and Counselling, Universitas Negeri Malang, Malang, Indonesia; 2 Department of Guidance and Counselling, Universitas Negeri Surabaya, Surabaya, Indonesia

Dear Editor,

We write in response to the important findings from the qualitative review entitled *‘Inclusive palliative care for LGBTQIA + individuals: A socioecological perspective on barriers and enablers’* published in *Palliative and Supportive Care* (Almeida-Godinho and Reis-Pina 2025). This study highlights that structural discrimination and the lack of cultural competence among healthcare professionals are major barriers to the provision of palliative care for LGBTQIA + individuals, underscoring the urgent need for systemic reform to foster inclusive and equitable care services.

The review emphasizes that individual, interpersonal, organizational, community, and policy dimensions interact to influence access to palliative care (Lewis et al. 2013). Conversely, although the findings reaffirm the importance of professional training and community engagement, the limited sample size and lack of representation of older adults or terminally ill patients raise concerns regarding the generalizability of the results (Gross et al. 2005). This gap suggests that further research is required to better understand the lived experiences of LGBTQIA + individuals facing serious illness and end-of-life challenges.

The study reviewed 55 participants, most of whom were young and urban residents. However, there was insufficient data to portray the experiences of older patients or those living in rural areas. Without representative data from older populations and communities with limited healthcare access particularly transgender or nonbinary individuals who face socioeconomic barriers the findings remain partial (Renner et al. 2021). The absence of these groups further reinforces the evidence gap regarding structural inequalities in palliative care. Without cross-contextual and longitudinal data, it is nearly impossible to build a universally applicable and culturally sensitive model of intervention.

Variability in the findings also reflects gaps in data collection across healthcare systems, especially in the areas of policy and professional training. Notably, the striking differences in perceptions between service providers and patients indicate that LGBTQIA + perspectives remain insufficiently integrated into healthcare training curricula (Ford et al. 2024).

To address these gaps, there is an urgent need to develop collaborative models between healthcare institutions and LGBTQIA + organizations to facilitate the exchange of knowledge and best practices. Such initiatives would enhance cultural literacy among healthcare professionals, expand access to diversity-affirming services, and foster the development of inclusive, evidence-based clinical practice guidelines (Day and Brömdal 2024).

The review also underscores the need for cross-national comparative studies, which remain limited to date. As global attention to equity and diversity in healthcare continues to grow, such research is crucial to map the social, legal, and cultural contexts that shape the implementation of inclusive palliative care.

In conclusion, this study reaffirms that inclusivity and cultural competence are fundamental to humane palliative care. However, the lack of data across age groups, social contexts, and healthcare systems continues to challenge the generalizability and applicability of existing findings. Therefore, we strongly encourage multidisciplinary collaborative research to strengthen empirical evidence and promote equity-based practice in the future.
